# Phosphatidylcholine synthesis through cholinephosphate cytidylyltransferase is dispensable in *Leishmania major*

**DOI:** 10.1038/s41598-019-44086-6

**Published:** 2019-05-20

**Authors:** Samrat Moitra, Mattie C. Pawlowic, Fong-fu Hsu, Kai Zhang

**Affiliations:** 1 Department of Biological Sciences, Texas Tech University, Lubbock, TX 79409 USA; 2 Wellcome Centre for Anti-Infectives Research (WCAIR), Division of Biological Chemistry and Drug Discovery, School of Life Sciences, University of Dundee, Dundee, DD1 5EH UK; 3 Department of Internal Medicine, Washington University School of Medicine, 660S. Euclid Ave., Saint Louis, MO 63110 USA

**Keywords:** Parasite biology, Phospholipids, Pathogens, Membrane lipids

## Abstract

Phosphatidylcholine (PC) is a major cell membrane constituent and precursor of important second messengers. In *Leishmania* parasites, PC synthesis can occur via the choline branch of the Kennedy pathway, the N-methylation of phosphatidylethanolamine (PE), or the remodeling of exogenous phospholipids. To investigate the role of *de novo* PC synthesis in *Leishmania major*, we focused on the cholinephosphate cytidylyltransferase (CPCT) which catalyzes the formation of CDP-choline, a key intermediate in the choline branch of the Kennedy pathway. Without CPCT, *L*. *major* parasites cannot incorporate choline into PC, yet the CPCT-null mutants contain similar levels of PC and PE as wild type parasites. Loss of CPCT does not affect the growth of parasites in complete medium or their virulence in mice. These results suggest that other mechanisms of PC synthesis can compensate the loss of CPCT. Importantly, CPCT-null parasites exhibited severe growth defects when ethanolamine and exogenous lipids became limited or when they were co-cultured with certain bacteria that are known to be members of sandfly midgut microbiota. These findings suggest that *Leishmania* employ multiple PC synthesis pathways to utilize a diverse pool of nutrients, which may be crucial for their survival and development in the sandfly.

## Introduction

Leishmaniases are a group of neglected tropical diseases transmitted through the bite of female phlebotomine sandflies^1^. The causative agents are protozoan parasites of the genus *Leishmania* which alternate between flagellated promastigotes colonizing the midgut of sandflies and non-flagellated amastigotes residing in the macrophages of mammals. Without a safe vaccine, disease management primarily depends on vector control and drugs^2^. Discoveries that reveal fundamental insights into *Leishmania* biology can lead to new drug targets, better treatments, and improved vector control strategies.

The plasma membrane of *Leishmania* parasites contains a combination of glycerophospholipids, sphingolipids, and ergostane-based sterols^3–6^. Besides being membrane components, these lipids play important roles in the anchoring of glycoconjugates and the formation of ordered membrane microdomains or lipid rafts^5,7–9^. *Leishmania* parasites are capable of synthesizing these lipids *de novo*. Enzymes involved in the biosynthesis of sphingolipids and sterols are often crucial for stress response and virulence^10–12^. In addition to *de novo* synthesis, *Leishmania* parasites also acquire lipids from the media (for promastigotes) or host (for amastigotes)^12–16^.

As in most eukaryotes, glycerophospholipids constitute the most abundant class of lipids in *Leishmania*^4,5^. The physical nature and function of glycerophospholipids are dictated by the charge of the head group, and the length and saturation of the fatty acyl chains that are attached to the glycerol backbone. The most abundant glycerophospholipid in *Leishmania* are phosphatidylcholine (PC) which constitutes 30–35% of total cellular lipids^5,17^. Because of its positively charged head group, PC is a membrane-forming phospholipid that is more abundant on the outer leaflet of the plasma membrane^18,19^. In mammalian cells, PC also functions as the precursor of several signaling molecules such as diacylglycerol, phosphatidic acid, and lyso-phospholipid^20^.

**Figure 1:**
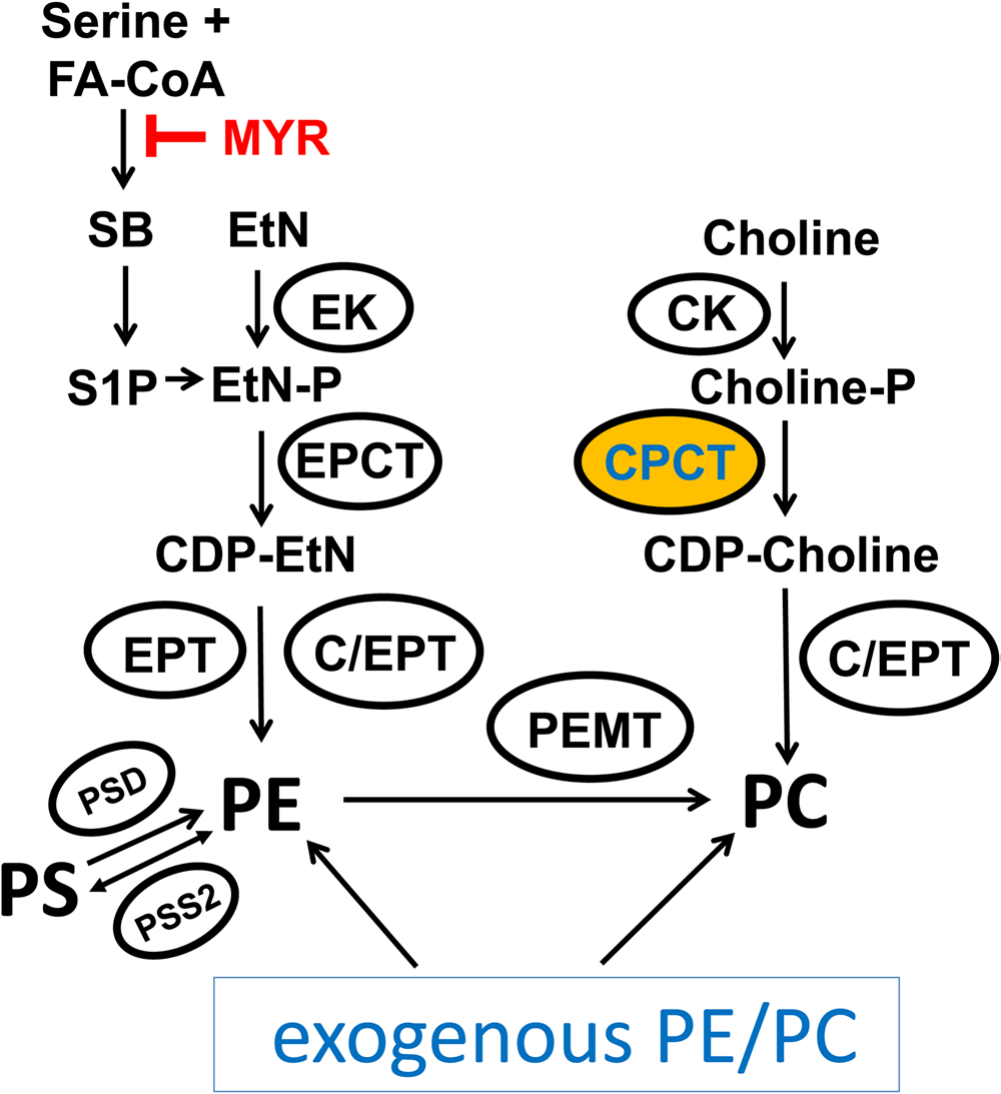
Predicted PE and PC synthesis in *Leishmania*. EK: ethanolamine kinase; EPCT: ethanolaminephosphate cytidylyltransferase; EPT: ethanolamine phosphotransferase; CK: choline kinase; CPCT: cholinephosphate cytidylyltransferase; C/EPT: choline/ethanolamine phosphotransferase; PEMT: phosphatidylethanolamine *N*-methyltransferase; PSD: phosphatidylserine decarboxylase; PSS2: phosphatidylserine synthase 2. EtN: ethanolamine; EtN-P: ethanolamine phosphate; PE: phosphatidylethanolamine; Choline-P: choline phosphate; PC: phosphatidylcholine; PS: phosphatidylserine; MYR: myriocin; S1P: sphingosine-1-phosphate; FA-CoA: fatty acyl-CoA.

In many eukaryotes including *Leishmania*, the *de novo* synthesis of PC starts with the phosphorylation of choline by choline kinase^21,22^; the resulting choline phosphate (choline-P) is then converted into CDP-choline by cholinephosphate cytidylyltransferase (CPCT); and finally, the enzyme choline/ethanolamine phosphotransferase (C/EPT) conjugates CDP-choline and diacylglycerol (DAG) into PC (Fig. 1). A similar pathway is responsible for the *de novo* synthesis of phosphatidylethanolamine (PE), as ethanolamine (EtN) is phosphorylated by ethanolamine kinase (EK), and the resulting ethanolamine phosphate (EtN-P) is converted into CDP-EtN by ethanolaminephosphate cytidylyltransferase (EPCT). In *Leishmania*, CDP-EtN is utilized to synthesize either plasmenylethanolamine (PME)^23^ or 1,2-diacyl-PE (Fig. 1). These two biosynthetic routes, collectively known as the Kennedy pathway^24^, is responsible for the production of the majority of PC and PE in many mammalian cell types and *Trypanosoma brucei*, a kinetoplastid parasite closely related to *Leishmania* species^22,25–28^.

In *Leishmania*, the sphingoid base metabolism can effectively convert serine into EtN-P which is then incorporated into PE^10,29^ (Fig. 1). Genomes of *Leishmania* species contain orthologs of phosphatidylserine synthase 2 and phosphatidylserine decarboxylase, suggesting that these parasites could generate PE through phosphatidylserine (Fig. 1). It is known that *Leishmania* parasites can also synthesize PC through the N-methylation of PE using S-adenosine-methionine as the methyl donor^23,30^. This PE N-methylation pathway is the predominant PC synthesis route in hepatocytes^31^ and *Saccharomyces cerevisiae*^32^, but is absent in *Trypanosoma brucei*^33,34^. Additionally, *Leishmania* can directly take up lipids including glycerophospholipids from the host or media and then remodel them into parasite-specific lipids^35–38^. So why would *Leishmania* parasites retain multiple, seemingly redundant PC synthesis pathways (Fig. 1)? What is the relative contribution of each pathway to the overall PC production during the promastigote and amastigote stages of *Leishmania*? And, does each mechanism favor the synthesis of particular PC species (variants in fatty acyl chain length and saturation)? Addressing these questions will provide novel insights into the physiological role of PC synthesis in *Leishmania* and may facilitate the development of new treatments.

In this study, we generated a CPCT-null mutant (*cpct*^*−*^) in *Leishmania major*, the etiological agent of cutaneous leishmaniasis in the old world^39^. CPCT is generally considered the rate-limiting enzyme in the *de novo* synthesis of PC^34,40,41^. In malaria parasites, CPCT is a vital enzyme for PC synthesis and a potential therapeutic target^42–44^. While *L*. *major cpct*^−^ mutants failed to incorporate choline into PC, they retained a similar level and composition of PC as wild type (WT) parasites when cultivated in complete media. Deletion of CPCT did not affect the proliferation of promastigotes in complete media or their virulence in mice. These findings indicate that *Leishmania* parasites can compensate the loss of *de novo* PC synthesis through other mechanisms such as PE N-methylation and lipid salvage. Importantly, *cpct*^−^ promastigotes did show significant growth reduction under starvation conditions when EtN and exogenous lipids became limited. Retaining the choline branch of the Kennedy pathway may allow *Leishmania* parasites to survive in the sandfly midgut when they must compete for nutrients with other microorganisms.

## Results

### Targeted deletion and cellular localization of CPCT in *L*. *major*

**Figure 2:**
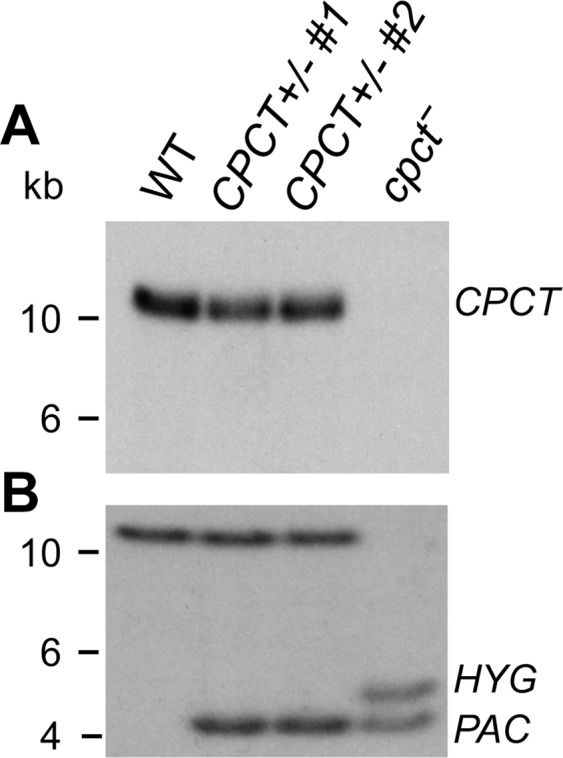
Southern blot confirms the targeted deletion of *CPCT*. Genomic DNA samples from WT, *CPCT*+/−, and *cpct*^−^ parasites were digested with restriction enzyme SacII and separated on agarose gels. Blots were probed with radiolabeled DNA fragments recognizing the ORF (**A**) or an upstream flanking region of *CPCT*. (**B**) The replacement of *CPCT* alleles by *PAC* and *HYG* is indicated. Full-size, unedited blots and loading controls are presented in Supplementary Fig. S1.

To explore the impact of *de novo* PC synthesis from choline in *L*. *major*, we focused on a CPCT ortholog (Tritrypdb ID: LmjF.18.1330, 592 amino acids) which is expected to catalyze the production of CDP-choline from CTP and choline-P (Fig. 1). *L*. *major* CPCT has six predicted transmembrane helices and no obvious N-terminal signal sequence. The endogenous *CPCT* alleles were deleted from *L*. *major* WT parasites and the resulting *cpct*^−^ mutants were confirmed by Southern blot (Fig. 2 and Fig. S1). The null mutant was then complemented with a plasmid to restore CPCT expression (*cpct*^−^/+*CPCT*).

**Figure 3:**
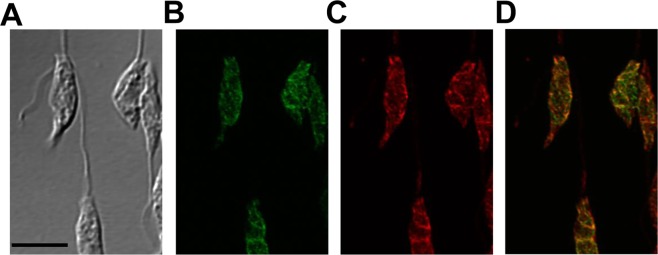
Endoplasmic Reticulum (ER) localization of CPCT in *L*. *major*. Log phase promastigotes of *cpct*^−^/+*GFP-CPCT* were labeled with rabbit anti-*T*. *brucei* BiP antiserum followed by a goat anti-rabbit IgG-Texas Red antibody and subjected to confocal immunofluorescence microscopy. (**A**) Phase contrast; (**B**) GFP fluorescence; (**C**) Anti-BiP staining; (**D**) Merge of B and C. Scale bar: 5 µm. The overlap between BiP and GFP-CPCT was determined by the JaCOP Image J analysis of 30 cells (Table S1).

In mammalian cells and yeast, CPCT is reported to relocate from nucleoplasm/cytoplasm to nuclear membrane and endoplasmic reticulum (ER) in response to the need for PC synthesis^45,46^. To examine the cellular localization of *L*. *major* CPCT, GFP-fusion proteins were introduced into the *cpct*^−^ mutant (Fig. S2). In fluorescence microscopy, both GFP-CPCT and CPCT-GFP exhibited a diffused, membranous pattern resembling the bulk ER (Fig. 3 and data not shown). Quantative analysis of GFP-CPCT showed ~85% overlap (by JaCOP Image J analysis of 30 randomly selected cells, Table S1) with the ER marker BiP^47,48^. Thus, CPCT is mainly located in the ER (Fig. 3 and Table S1).

### *Cpct*^−^ mutants cannot synthesize PC from choline but can incorporate EtN into PE and PC

**Figure 4:**
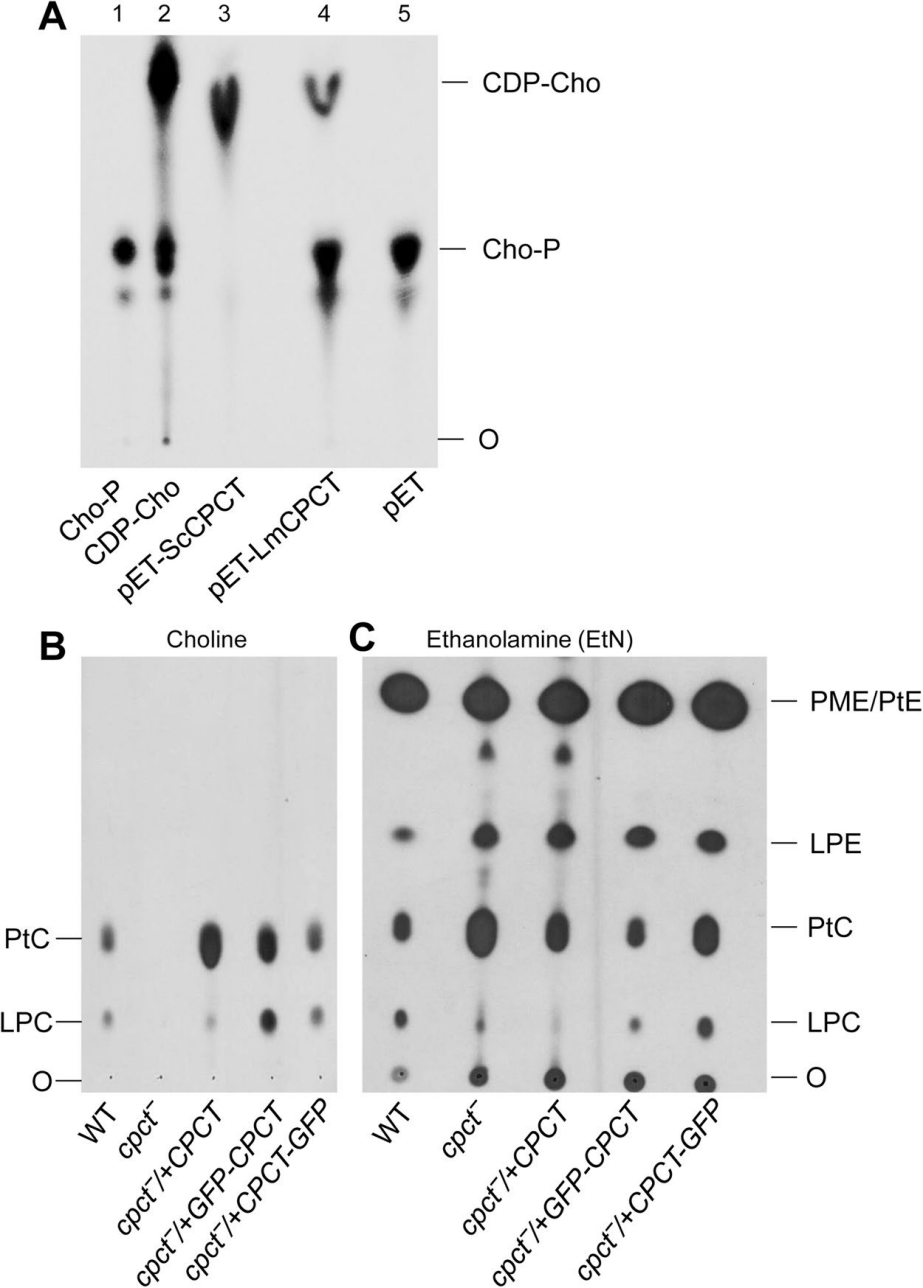
*CPCT* is required for incorporating choline to PC. (**A**) Lane 1: [^14^C]-Choline-P. Lane 2: [^14^C]-CDP-choline. Lane 3–5: cell lysates from *E*. *coli* transformed with pET-*Sc*CPCT, pET-*Lm*CPCT, or pET only were incubated with [^14^C]-labeled choline-P, followed by TLC analysis as described in METHODS. (**B**,**C**) Log phase *Leishmania* promastigotes were cultivated in the presence of [^3^H]-labeled choline (**B**) or [^3^H]-labeled EtN (**C**). Total lipids were extracted after 48 hours and analyzed by TLC. O: origin of loading; LPC: lyso-phosphatidylcholine; LPE: lyso-phosphatidylethanolamine. Full-size, unedited images are presented in Supplementary Fig. S4.

To determine whether CPCT is required for the synthesis of CDP-choline, *E*. *coli* lysates containing recombinant *L*. *major* CPCT (*Lm*CPCT) or *S*. *cerevisiae* CPCT (*Sc*CPCT) were incubated in the presence of CTP and radiolabeled choline-P and the products were examined by thin layer chromatography (TLC). As shown in Fig. 4A and Fig. S3A, lysate containing *Sc*CPCT could efficiently catalyze the formation of CDP-choline whereas lysate from empty pET vector had no activity. By comparison, *Lm*CPCT conferred a low but clearly detectable level of CPCT activity (~26% of *Sc*CPCT, Fig. 4A and Fig. S3A). It is not clear whether this result reflects the intrinsic difference between *Sc*CPCT and *Lm*CPCT in specific activity, or their ability to be functionally expressed in *E*. *coli*.

In agreement with this finding, *L*. *major* WT parasites but not *cpct*^−^ mutants could incorporate [^3^H]-choline into PC including 1,2-diacyl-PC (PtC) and lyso-PC (LPC, a hydrolytic product of PtC^49^) (Fig. 4B and Fig. S3B). Complementation of *cpct*^−^ with CPCT, GFP-CPCT or CPCT-GFP led to robust assimilation of choline into PtC and LPC (Fig. 4B and Fig. S3B). These findings indicate that CPCT is solely responsible for generating PC from choline in *L*. *major*. As we reported previously^23^, WT parasites were able to incorporate [^3^H]-EtN into PE (PME + 1,2-diacyl-PE + lyso-PE or LPE^49^) and PC (Fig. 4C and Fig. S3C); while similar results were observed with the *cpct*^−^ mutants, we detected a 1.5–1.9-fold increase in the incorporation of [^3^H]-EtN into PC (Fig. 4C and Fig. S3C), which could be a compensatory response to the loss of PC synthesis from choline.

### *Cpct*^−^ mutants contain normal levels of PC and PE when grown in the complete medium

**Figure 5:**
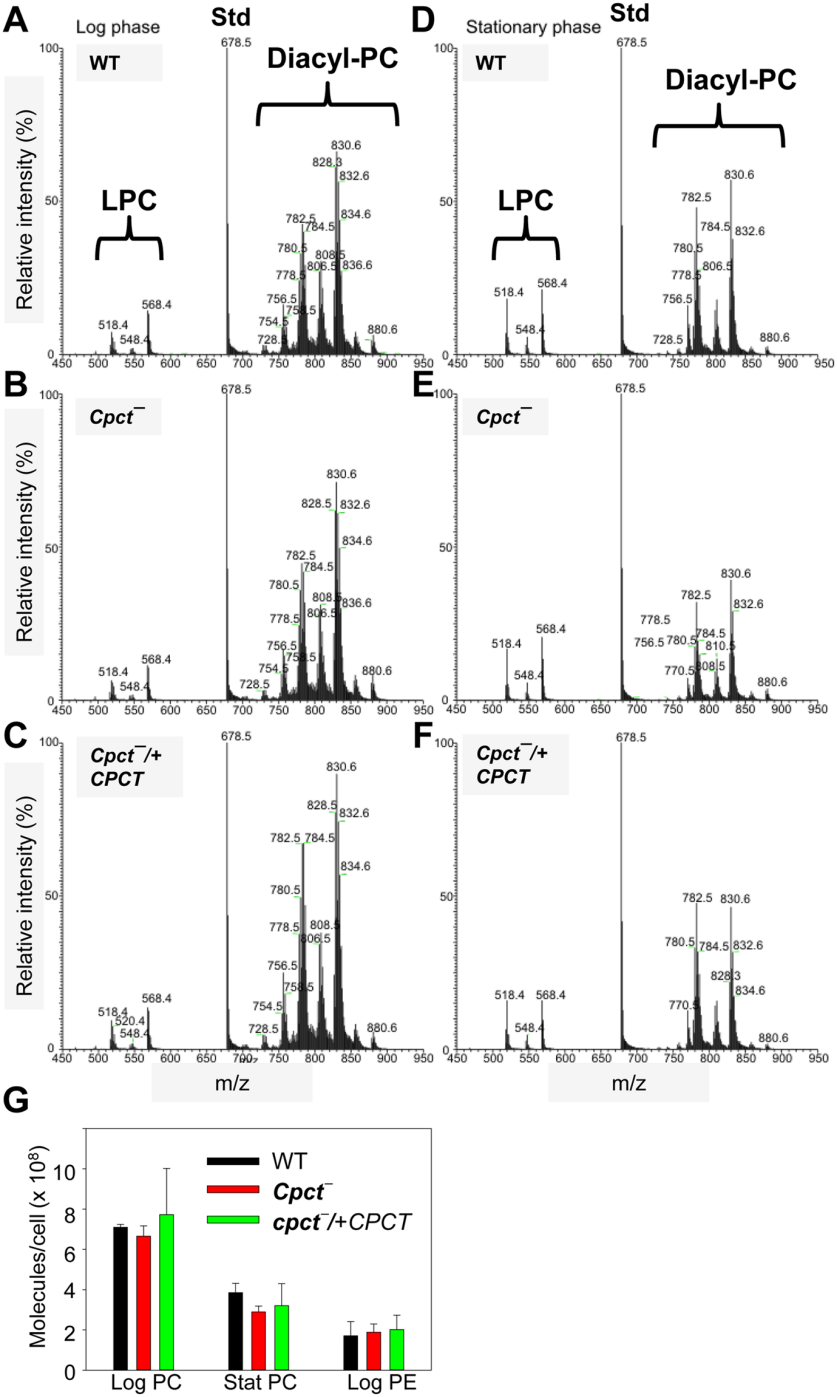
*Cpct*^−^ mutants contain normal levels of PC. Total lipids from log phase (**A**–**C**) and stationary phase (**D**–**F**) promastigotes were examined by ESI/MS in the positive ion mode. (**A**,**D**): WT; (**B**,**E**): *cpct*^−^; (**C**,**F**): *cpct*^−^/+*CPCT*. Peaks corresponding to LPC and diacyl-PC are indicated. Std: PC standard (14:0/14:0-PC) for quantitation. (**G**) Abundance of PC (diacyl-PC + LPC) and PE in *L*. *major* promastigotes were determined and averaged from three independent experiments. Error bars represent standard deviations from three biological repeats.

To examine whether the choline branch of Kennedy pathway plays a major role in the overall PC production in *L*. *major*, we extracted total lipids from promastigotes cultured in the complete M199 medium (M199 medium with 10% fetal bovine serum or FBS and other supplements)^50^. The composition of PC was assessed using electrospray ionization mass spectrometry (ESI/MS) in the positive-ion mode. As shown in Fig. 5A–F, in log phase and stationary phase, the LPC and diacyl-PC species in *cpct*^−^ mutants closely resembled those found in WT and *cpct*^−^/+*CPCT* parasites. Through comparison with a PC standard, we estimated the overall abundance of PC in log phase *cpct*^−^ mutants to be 6.5–7.1 × 10^8^ molecules/cell, which was close to the average values in log phase WT and *cpct*^−^/+*CPCT* parasites (Fig. 5G). During the stationary phase, *cpct*^−^ mutants contained 2.6–3.1 × 10^8^ PC molecules/cell, whereas WT and *cpct*^−^/+*CPCT* parasites had 3–4 × 10^8^ molecules/cell (Fig. 5G; the difference between WT and *cpct*^−^ is not statistically significant). The decrease of PC abundance in stationary phase is consistent with the global lipid remodeling when promastigotes transition from replicative procyclics to infectious metacyclics^10,51^.

In addition to PC, we examined the cellular levels of PE in log phase promastigotes. As summarized in Fig. 5G, no significant difference was detected between WT, *cpct*^−^ and *cpct*^−^/+*CPCT* parasites (1.7–2.1 × 10^8^ PE molecules/cell). Thus, the choline branch of Kennedy pathway is not required for bulk phospholipid synthesis when *L*. *major* parasites were cultured in the complete medium. These results suggest that *Leishmania* promastigotes can compensate the loss of CPCT through PE N-methylation and/or lipid uptake followed by remodeling to meet the demand of PC synthesis.

### Role of CPCT in *Leishmania* differentiation and growth under EtN-limiting conditions

**Figure 6:**
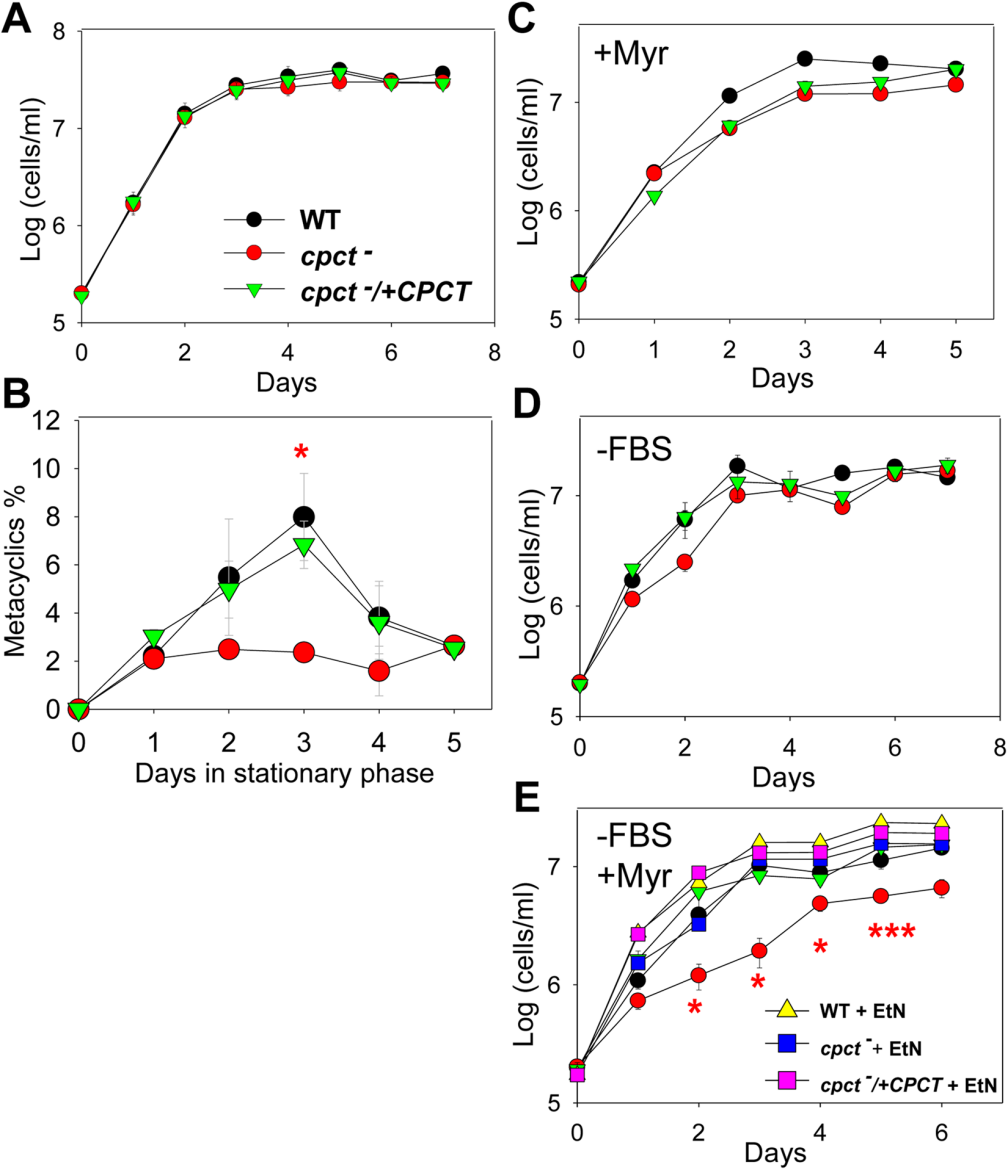
*CPCT*-null mutants proliferate normally in the complete medium but show significant growth delay under EtN-limiting conditions. Promastigotes were cultivated under various conditions and culture densities were determined daily using a hemocytometer in A, C-E. In B, percentages of metacyclics during stationary phase were determined daily as described in METHODS. Culture conditions: (**A**,**B**) complete M199 medium; (**C**) complete M199 medium + 4 μM of myriocin; (**D**) lipid-free M199 medium (no FBS); and (**E**) lipid-free M199 + 4 μM of myriocin with or without 250 μM of EtN. Error bars represent standard deviations from three independent experiments (*p < 0.05; ***p < 0.001).

When cultivated in the complete medium, *cpct*^−^ mutants grew and replicated at a similar rate as WT and *cpct*^−^/+*CPCT* parasites (Fig. 6A). These mutants produced less metacyclics (the infective form to mammals)^52^ than WT and add-back parasites during the stationary phase (Fig. 6B). In *L*. *major*, formation of metacyclics (metacyclogenesis) is associated with the modification of lipophosphoglycan (LPG)^53^. Western blot analysis revealed that LPG in stationary phase *cpct*^−^ was of normal abundance, but migrated slightly slower than that from WT and *cpct*^−^/+*CPCT* parasites (Fig. S4), suggesting minor structural alteration of LPG.

To further examine the contribution of CPCT *in vitro*, we monitored the growth of *cpct*^−^ mutants under various EtN-limiting conditions. When promastigotes were inoculated in complete M199 medium in the presence of myriocin, which inhibits the conversion of serine into EtN-P (Fig. 1), no major growth defects were observed in the *cpct*^−^ mutants (Fig. 6C). This suggests that uptake of exogenous PE and/or PC is sufficient to compensate the loss of CPCT (Fig. 1). Similarly, in a lipid-free M199 medium (M199 without FBS but with 0.4% fatty acid free bovine serum albumin), *cpct*^−^ mutants proliferated nearly as well as WT and *cpct*^−^/+*CPCT* parasites showing a slight but not statistically significant delay (Fig. 6D). This suggests that in the absence of lipid uptake, the serine-to-EtN conversion is largely sufficient for PC synthesis (Fig. 1). However, when inoculated in lipid-free M199 (no exogenous lipids available to uptake) containing myriocin (inhibiting serine-to-EtN conversion), *cpct*^−^ mutants grew significantly slower than WT and *cpct*^−^/+*CPCT* parasites (Fig. 6E). Finally, supplementation of EtN rescued the growth of *cpct*^−^ to WT levels under this condition (Fig. 6E). Together, these results suggest that in comparison to WT and *cpct*^−^/+*CPCT* parasites, *cpct*^−^ mutants are more dependent on PE N-methylation and the uptake of exogenous lipids for PC synthesis.

### *Cpct*^−^ mutants do not have virulence defects in mice

**Figure 7:**
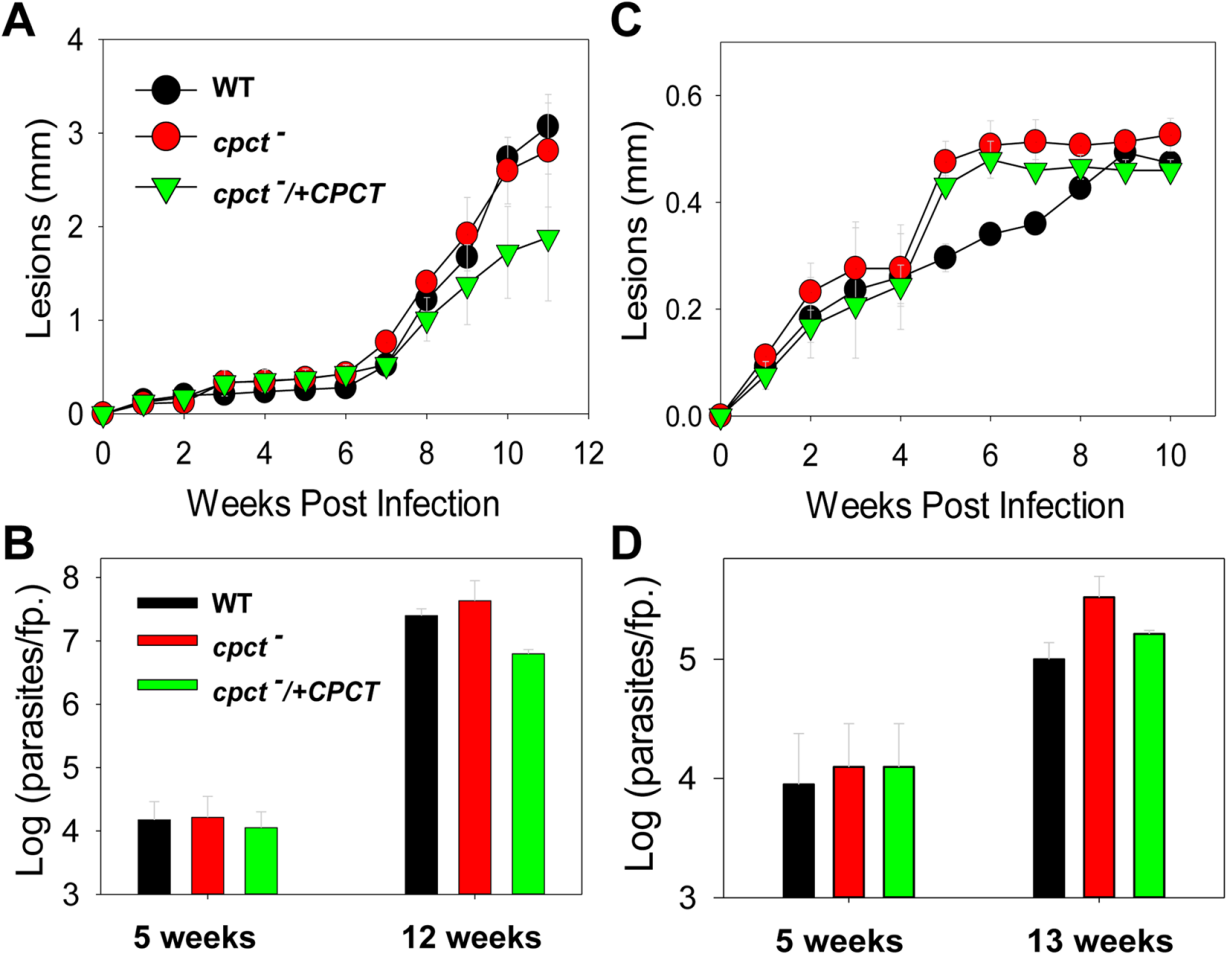
*Cpct*^−^ mutants are fully virulent. BALB/c mice (**A**,**B**) and C57BL/6 mice (**C**,**D**) were infected with stationary phase promastigotes as described in METHODS. Footpads lesions were measured weekly and plotted in (**A**,**C**). Limiting dilution assay was performed at the indicated times to determine parasite burden (**B**,**D**). Error bars represent standard deviations (5 mice per group).

To study the role of *de novo* PC synthesis in *Leishmania* virulence, mice were infected subcutaneously in the footpad with late stationary phase WT, *cpct*^−^, and *cpct*^−^/+*CPCT* promastigotes. In both BALB/c mice (susceptible to *L*. *major*; Fig. 7A) and C57BL/6 mice (resistant to *L*. *major*; Fig. 7C)^54^, *cpct*^−^ mutants induced lesions of similar sizes over time as WT and *cpct*^−^/+*CPCT* parasites. Limiting dilution assay was performed at 5, 12, or 13 weeks post infection to determine parasite burden (Fig. 7C-D). No significant difference was observed between WT and *cpct*^−^ parasites. Therefore, although CPCT affects metacyclogenesis *in vitro* (Fig. 6B), it is not required for the virulence of *L*. *major*, suggesting that amastigotes can fulfill their need for PC synthesis through lipid salvage or PE N-methylation (Fig. 1).

### *Cpct*^−^ mutants show growth delay when co-cultured with certain bacteria

**Figure 8:**
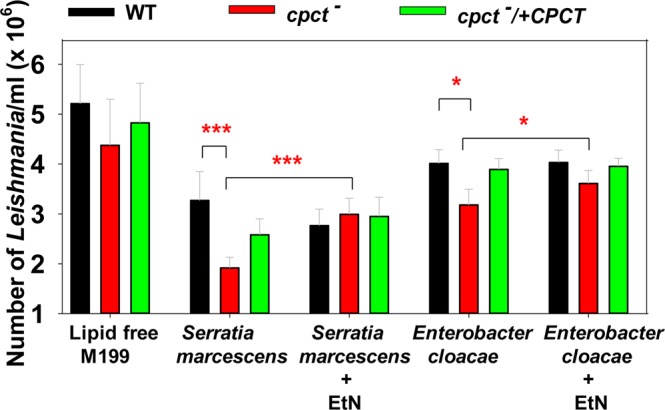
*Cpct*^−^ mutants show significant growth delay when co-cultured with bacteria and the growth delay can be rescued by EtN supplementation. Effects of *Serratia marcescens* and *Enterobacter cloacae* on the growth of *cpct*^−^ mutants were determined in a co-culture experiment. *L*. *major* promastigotes (1 × 10^6^ cells/ml) were incubated in lipid-free M199 medium alone or with *Serratia marcescens* (50 bacteria/ml) or *Enterobacter cloacae* (50 bacteria/ml). EtN (250 μM) was included as indicated. The number of promastigotes/ml was recorded using a hemocytometer after 24 h. Results were averaged from three independent experiments with triplicates. Error bars represent standard deviations from three biological repeats (*p < 0.05; ***p < 0.001) based on one way Anova relative to WT.

In the midgut of sandfly, *Leishmania* promastigotes need to proliferate and differentiate in the presence of a community of microorganisms^55–59^. The interaction between parasites and sandfly microbiota has significant impact on *Leishmania* differentiation and transmission^55,60,61^. While PC is the most abundant phospholipid in eukaryotes, most bacteria synthesize PE as a major membrane lipid and have a high demand for EtN^62–65^. Here we examined whether CPCT was required when *L*. *major* promastigotes were co-cultured with *Serratia marcescens* or *Enterobacter cloacae*, two Gram-negative bacteria that have been identified in the midgut of several sandfly species^59,66,67^. In this experiment, promastigotes (1.0 × 10^6^ cells/ml) and bacteria (50 cells/ml) were co-cultured in lipid-free M199 media and the density of *Leishmania* parasite was determined after 24 hours. As shown in Fig. 8, all parasites grew slower in the presence of *S*. *marcescens* presumably due to nutrient competition and waste/toxin production from the bacteria. For WT parasites, a 37% reduction was observed during *S*. *marcescens* co-culture, while *cpct*^−^ and *cpct*^−^/+*CPCT* mutants displayed 61% and 45% reduction respectively (Fig. 8, lipid free M199 vs. *S*. *marcescens*). Thus, losing CPCT seems to reduce parasites’ fitness under a competitive condition. Importantly, addition of EtN largely restored the replication of *cpct*^−^ to levels close to WT and *cpct*^−^/+*CPCT* parasites (Fig. 8, *S*. *marcescens* vs. *S*. *marcescens* + EtN). By comparison, a less significant effect on *Leishmania* growth was observed during co-culture with *Enterobacter cloacae*, although *cpct*^−^ mutants still replicated slower than WT and add-back parasites, and EtN supplementation improved their growth (Fig. 8, *E*. *cloacae* vs. *E*. *cloacae* + EtN). Together, these findings suggest that the choline branch of the Kennedy pathway allows *Leishmania* to be less dependent on EtN, which may be a limiting nutrient in the sandfly (Fig. 1).

## Discussion

In this study, we characterized an ER-localized CPCT that is responsible for the incorporation of choline into PC in *L*. *major*. Deletion of CPCT had no obvious impact on the cellular levels of PC or PE when promastigotes were cultivated in complete M199 media (Fig. 6). *Cpct*^−^ mutants replicated normally and did not show any defects in morphology when they were able to generate PC through PE N-methylation and/or lipid salvage. We observed a 2–4 folds reduction in metacyclogenesis during late stationary phase (Fig. 6B), which may be related to the minor alteration of LPG in *cpct*^−^ (Fig. S4)^53,68^. Importantly, the fact that of *cpct*^−^ mutants are fully virulent and replicative mice (Fig. 7) suggests that the *de novo* synthesis of PC is not required during the mammalian stage of *L*. *major*.

Meanwhile, the proliferation of *cpct*^−^ mutants was severely reduced when they were cultivated in a lipid-free medium (which eliminated the uptake, degradation and remodeling of exogenous lipids) containing myriocin (which blocked the conversion of serine into EtN-P via sphingoid base metabolism) (Fig. 6). These findings indicate that the choline branch of the Kennedy pathway is dispensable when parasites can synthesize PC from EtN, serine, or exogenous lipids (Fig. 1).

The fact that *cpct*^−^ mutants can still grow (albeit at a reduced rate) and synthesize PC in lipid-free M199 containing myriocin (Fig. 6D) suggests that residue amount of EtN/EtN-P (which may be converted from serine after myriocin loses efficacy) is sufficient to sustain a low level of PC synthesis and cell proliferation. Alternatively, parasites may generate PE/EtN from serine via the activity of phosphatidylserine synthase 2 and phosphatidylserine decarboxylase^69^ (Fig. 1). These two enzymes are essential for the optimal growth and mitochondrial function of procyclic *Trypanosoma brucei*, and their exact roles in *Leishmania* have yet to be determined^69^.

Since *cpct*^−^ mutants are fully replicative and virulent in mice (Fig. 7), we postulate that the ability to synthesize PC from choline is dispensable during the mammalian stage when amastigotes reside within the phagolysosome of macrophages and have access to lipids, amino acids, sugars and amino sugars^70–72^. Meanwhile, it is of interest to determine if CPCT contributes to the proliferation of *Leishmania* promastigotes in the midgut of sandfly, where they must compete for nutrients with the resident microbiota^55,73^ (Fig. 8). While the exact composition of carbon source in the sandfly gut is not well defined, the diet of female sandfly consists of blood meals and nectar, which is digested by the hydrolytic enzymes from the sandfly and microbiota. Because EtN or PE is an important source of carbon and nitrogen for many bacterial species^64,65^, these nutrients may be limited in the sandfly gut. In contrast, choline is not an essential nutrient for many bacteria which mainly synthesize PE, phosphatidylglycerol (PG), and cardiolipin as their membrane phospholipids^74^. Retaining the choline branch of the Kennedy pathway may allow *Leishmania* to synthesize PC from choline when EtN/exogenous phospholipid is limited. In support of this hypothesis, *cpct*^−^ mutants showed a significant growth delay when co-cultured in a lipid-free medium with *Serratia marcescens* or *Enterobacter cloacae*, two bacteria found in both field-captured and lab-reared sandflies, and the supplementation of EtN largely restored the proliferation of *cpct*^−^ mutants (Fig. 8). The reason why *E*. *cloacae* has less inhibitory effect on *Leishmania* than *S*. *marcescens* may involve a combination of factors including their rates on EtN consumption, nutrient depletion and toxin production.

In summary, *L*. *major* promastigotes may retain the choline branch of the Kennedy pathway to survive nutrient-limiting conditions. Such metabolic flexibility may improve their competitive fitness in the sandfly midgut.

## Methods

### Materials

Ethanolamine (EtN) hydrochloride ([1-^3^H], 40–60 Ci/mmol) and [methyl-^14^C] phosphorylcholine (50–60 mCi/mmol) were purchased from American Radiolabeled Chemicals (St. Louis, MO). Choline chloride [methyl-^14^C] (50–60 mCi/mmol) and cytidine diphosphocholine [methyl-^14^C] (50–60 mCi/mmol) were purchased from Perkin Elmer, Inc (Waltham, MA). Lipid standards for mass spectrometry including 1,2-dipalmitoyl-*sn*-glycero-3-phosphoethanolamine (16:0/16:0-PE) and 1,2-dimyristoyl-*sn*-glycerol-3-phosphocholine (14:0/14:0-PC) were purchased from Avanti Polar Lipids (Alabaster, AL). Fatty acid free, low endotoxin bovine serum albumin was purchased from Sigma-Aldrich (St. Louis, MO). Antisera for mouse-anti-alpha tubulin, rabbit-anti-GFP, goat-anti-rabbit-IgG-HRP, and goat-anti-mouse-IgG-HRP were purchased from Life Technologies (Carlsbad, CA). All other reagents were purchased from VWR International (Radnor, PA) or Thermo Fisher Scientific (Hampton, NH) unless specified otherwise.

### Molecular cloning

With the exception of cloning *Saccharomyces cerevisiae CPCT* into pET vector (described below), CPCT refers to *L*. *major* CPCT in this study. To facilitate CPCT assay, the ORF of *Saccharomyces cerevisiae CPCT* (YGR202C) was amplified from yeast genomic DNA by PCR using primers 5′-GTACTGGGATCCATGGCAAACCCAACAACAGGGAAGTCC-3′/5′- GATCATGGATCCCAGTTCGCTGATTGTTTCTTCTTC-3′. The resulting 1280 bp DNA fragment was digested with BamHI and ligated into *E*. *coli* expression vector pET23a + to generate pET-*Sc*CPCT. The ORF of *L*. *major* CPCT was cloned into pET23a + to generate pET23a-*Lm*CPCT. These pET constructs were transformed into *E*. *coli* BL21 (DE3) competent cells.

The open reading frame (ORF) of *L*. *major* CPCT (LmjF18.1330) was amplified by PCR from *L*. *major* WT genomic DNA using primers 5′-GTACTGGGATCCACCATGCCGCATCTGCTTGAGCAC-3′/5′-GATCAGGGATCCTCATGCCCCGTCCTTCACC-3′. The resulting 1.8 kb DNA fragment was digested with BamHI and ligated into pXG1^75^, generating pXG1-CPCT. To study localization, the *CPCT* ORF was amplified and cloned into pXG1-GFP’ or pXG1-‘GFP to generate pXG1-GFP-CPCT or pXG1-CPCT-GFP, respectively.

To generate *CPCT* knockout constructs, the upstream and downstream sequences of *CPCT* ORF (~1 kb each) were amplified using primers 5′-GATCATGAGCTCAACGAACGGTAGCCCGCATATTG-3′/5′-GTCAGAACTAGTGATCTAGGATCCGGCAGACTCGTGTGTGTATG-3′ and 5′-GTCAGTGGATCCGGGGAGATGCCGGCGTCTTC-3′/5′-GTACGAAAGTCCAACGCCATCAACTATGCG-3′, respectively. The amplified upstream and downstream sequences were cloned in tandem into the cloning vector pUC18. Subsequently, genes conferring resistance to hygromycin (HYG) and puromycin (PAC) were cloned between these regions to generate pUC18-KO-CPCT: HYG and pUC18-KO-CPCT: PAC.

All the molecular constructs were verified by restriction enzyme digestion and DNA sequencing.

### *Leishmania* promastigote culture and genetic manipulation

Unless specified otherwise, *L*. *major* LV39 clone 5 (Rho/Su/59/P) promastigotes were cultured at 26 °C in M199 media with or without 10% heat-inactivated FBS and other supplements^50^. Cell density was determined using a hemocytometer. Percentages of metacyclics in stationary phase culture were determined as previously described^68^. To investigate the functions of CPCT, the endogenous *CPCT* alleles were deleted from *L*. *major* LV39 wild type (WT) parasites by two consecutive rounds of targeted replacement as described^50^ using linearized knockout constructs from pUC18-KO-CPCT:PAC and pUC18-KO-CPCT:HYG. The resulting *cpct*^−^ mutants (∆*CPCT::PAC*/∆*CPCT::HYG*) were confirmed by Southern blot, where genomic DNA from promastigotes was digested with *SacII*, separated on a 0.7% agarose gel, transferred to a nitrocellulose membrane, and probed with [^32^P]-labeled DNA fragments corresponding to the ORF or an upstream region of *CPCT*. Results of Southern blot were visualized by autoradiography. To restore *CPCT* expression, pXG1-CPCT was transfected into *cpct*^−^ mutants and referred to as *cpct*^−^/+*CPCT* (∆*CPCT::PAC*/∆*CPCT::HYG*/+pXG1-CPCT). For localization studies, pXG1-GFP-CPCT or pXG1-CPCT-GFP was introduced into *cpct*^−^ to generate *cpct*^−^/+*GFP-CPCT* or *cpct*^−^/+*CPCT-GFP*, respectively.

To examine cell growth under various nutrient-limiting conditions, we prepared M199 media that are supplemented with either 10% FBS (complete medium), 0.4% fatty acid free bovine serum albumin (lipid-free M199 medium, which contains 23–24 μM of serine but no EtN), 0.4% bovine serum albumin plus 4 μM of myriocin, or 0.4% bovine serum albumin plus 4 μM of myriocin and 250 μM of EtN. At low densities (<1.0 × 10^6^ cells/ml), cells were concentrated 10-fold before counting, and at high densities (>1 × 10^7^ cells/ml), cells were passed through a 27 ½ G needle three times to break up clumps before counting. To measure the percentage of dead cells parasites were labeled with 5.6 µg/ml propidium iodide followed by flow cytometry.

### Metabolic labeling, CPCT assay and thin layer chromatography (TLC)

To examine the incorporation of choline and EtN into phospholipids, promastigotes were inoculated into complete M199 media at 2.0 × 10^5^ cells/ml and labeled with 1 µCi/ml of choline chloride [methyl-^14^C] or 1 µCi/ml of EtN hydrochloride [1-^3^H]. Total lipids were extracted after 48 hours and dissolved in chloroform:methanol (1:2, v/v) at 2.0 × 10^9^ cells/ml. The incorporation of EtN or choline into *Leishmania* lipids was determined by scintillation counting, followed by one dimensional TLC (each lane contained lipid from 1.0 × 10^7^ cells) performed in a solvent made of methyl acetate:1-propanol:chloroform:methanol:0.9% KCl (25:25:25:10:9 by volume). The TLC plates were sprayed with EN^3^HANCE (Perkin Elmer, for ^3^H-EtN labeling) and exposed to autoradiography film. To quantify the incorporation of [^3^H]-EtN into PC over total [^3^H]-EtN incorporation into PE + PC, TLC images were subjected to densitometry analysis by Image J and results from two experiments were averaged.

To examine the activity of recombinant CPCT, *E*. *coli* BL21 (DE3) transformants containing pET23-*Lm*CPCT, pET23-*Sc*CPCT, or empty pET23a + vector were grown in LB media until OD_600 nm_ reached 0.6 and induced with 1 mM of IPTG for 3 hours. Bacteria were resuspened in a lysis buffer (Tris HCl 30 mM at pH7.4, 5% glycerol, 1 mM EDTA, and 1 x protease inhibitor) and sonicated on ice. Protocol of CPCT assay was adapted from a previous report^76^. Briefly, *E*. *coli* lysate containing 60 μg of bacterial protein was incubated in a 20 μl reaction mix containing 25 mM of MgCl_2_, 5 mM of CTP, and 0.1 mM of phosphorylcholine [methyl-^14^C] for 30 minutes at 30 °C. Reaction mix (10 μl each) was then analyzed by TLC using silica-60 plates and a solvent made of ethanol:0.5% NaCl:25% ammonium hydroxide (10:10:1 by volume). Radioactive signals were detected using a Personal Molecular Imager (Bio-Rad).

### Fluorescence microscopy

Promastigotes expressing GFP-CPCT or CPCT-GFP were attached to poly-L-lysine coated coverslips, fixed with 3.7% formaldehyde, and then permeabilized on ice with ethanol. Incubation with rabbit anti-*T*. *brucei* BiP antiserum (1:1000) was performed at room temperature for 40 minutes. After washing, coverslips were incubated with a goat anti-rabbit-Texas Red (1:2000) antiserum for 40 minutes. An Olympus Fluoview FV3000 Laser Scanning Confocal Microscope was used to visualize the expression and localization of GFP-CPCT. To quantify the overlap between GFP-CPCT and anti-BiP staining, 30 randomly selected cells were analyzed using Image J JACoP (Just Another Colocalization Plugin)^48^.

### Lipidomic analysis

In this study, all choline glycerophospholipids including 1,2-diacyl-phosphatidylcholine (PtC) and 1-acyl-phosphatidylcholine (lyso-PC) are referred to as PC. Similarly, all ethanolamine glycerophospholipids including plasmenylethanolamine (PME), 1,2-diacyl-phosphatidylethanolamine (PtE) and 1-acyl-phosphatidylethanolamine (lyso-PE) are referred to as PE.

To analyze composition and quantity of glycerophospholipids, promastigote lipids were extracted (1 × 10^8^ cells per sample) using the Bligh-Dyer approach^77^ and dissolved in a mixture of chloroform:methanol (1:2, v/v). Lipid standards were added to cell lysates prior to lipid extraction (5.0 × 10^7^ molecules/cell for 14:0/14:0-PC and 1.0 × 10^8^ molecules/cell for 16:0/16:0-PE; these lipids are not present in *L*. *major* promastigotes). Lipid samples were analyzed by electrospray ionization mass spectrometry (ESI/MS) as described^23^. Quantitation of PC and PE was performed using precursor ion scan of [*m*/*z*]^+^ 184 (positive ion mode) and [*m*/*z*]^−^ 196 (negative ion mode), respectively. All lipidomic analyses were performed three times.

### Western blot

Whole cell lysates from *Leishmania* promastigotes were resolved by SDS-PAGE, followed by immunoblotting with rabbit anti-GFP antibody (1:1000), anti-LPG monoclonal antibody WIC79.3 (1:1000) or monoclonal antibody against tubulin (1:5000), followed by appropriate secondary antibodies as previously described^78^. Signals from Western blot were quantified using a FluorChem E system (Protein Simple).

### Mouse infection

Use of mice in this study was approved by the Animal Care and Use Committee at

Texas Tech University (US PHS Approved Animal Welfare Assurance NO. A3629-01). BALB/c and C57BL/6 mice (female, 8 weeks old) were purchased from Charles River Laboratories International. Mice were housed and cared for in the facility operated by the Animal Care and Resources Center at Texas Tech University adhering to the Guide for the Care and Use of Laboratory Animals (the 8th Edition, NRC 2011) for animal husbandry. To maintain virulence, promastigotes were injected into the footpads of BALB/c mice and recovered after 3–4 weeks to start low passage *in vitro* cultures. To assess virulence, day 3 stationary phase promastigotes (cultured for less than five passages after recovery from mice) were resuspended in DMEM and injected into the left hind footpads of mice (1.0 × 10^6^ cells per mouse, 5 mice per group). Lesion sizes were measured weekly using a Vernier caliper and parasite loads were determined by limiting dilution assay^79^.

### *Leishmania* co-culture with bacteria

*Serratia marcescens* (ATCC #13880) and *Enterobacter cloacae* (Carolina Biological # 155032) were cultured in LB media at 37 °C until OD_600 nm_ reached 0.6. Bacteria were counted manually using a hemocytometer and diluted in lipid-free M199. For *Leishmania* bacteria co-culture, promastigotes were inoculated into lipid-free M199 at 1.0 × 10^6^ cells/ml with 50 bacteria/ml in 12-well plates (in the presence or absence of 250 μM of EtN) and incubated at 27 °C. Parasites cultured in the absence of bacteria were included as a control. Concentration of promastigotes was determined using a hemocytometer after 24 hours.

### Statistical analysis

Unless otherwise specified, all experiments (in Figs 5–8) were repeated three times and each biological repeat contained 2–3 technical repeats. Differences among experimental groups were determined by the unpaired Student’s *t* test (for two groups) or one way ANOVA (for three to four groups) using Sigmaplot 11.0 (Systat Software Inc, San Jose, CA). *P* values indicating statistical significance were grouped into values of <0.05 (*), <0.01 (**) and <0.001 (***).

## Supplementary information

Supplementary figures and tables
