# Supplementary material for: Phosphatidylcholine synthesis through cholinephosphate cytidylyltransferase is dispensable in Leishmania major
Source: Sci Rep. 2019 May 20;9:7602. doi: 10.1038/s41598-019-44086-6 (PMC6527706; doi:10.1038/s41598-019-44086-6)
Supplement: Supplementary file 1 — Supplementary figures and tables [file 41598_2019_44086_MOESM1_ESM.pdf]

Supplementary figures and tables

Manuscript title: Phosphatidylcholine synthesis through cholinephosphate cytidylyltransferase is dispensable in *Leishmania major*

Authors: Samrat Moitra, Mattie C. Pawlowic, Fong-fu Hsu, and Kai Zhang

Supplementary figure legends

Fig. S1. Southern blot confirms the targeted deletion of *CPCT* (full-size, unedited images for Figure 2). (**A**) 8-hour exposure for the blot hybridized with the *CPCT* ORF probe. (**B**) Ethidium bromide staining of the DNA gel used in **A**. (**C**) 48-hour exposure for the blot hybridized with the *CPCT* FR probe. (**D**) Ethidium bromide staining of the DNA gel used in **C**.

Fig. S2. Validation of GFP-tagged CPCT fusion proteins by Western blot. Cell lysates from log phase WT, WT/+ GFP (27 kDa), *c14dm⁻/+c14DM-GFP* (81 kDa), *cpct⁻/+GFP-CPCT* (92 kDa) and *cpct ⁻/+CPCT-GFP* (92 kDa) parasites were analyzed by Western blot using antibodies against GFP (**A**, 5-second exposure) or α-tubulin (**B**, 5-second exposure).

Fig. S3. *CPCT* is required for incorporating choline to PC (full-size, unedited images for Figure 4). (**A**) 10-hour exposure (Bio-Rad Personal Molecular Imager) of the CPCT assay using *E. coli* lysates (boiled and not boiled). (**B-C**) 24-hour exposure (autoradiography) of TLC analyses of metabolic labeling with [^{14}C]-labeled choline (**B**) or [^{3}H]-labeled EtN (**C**).

Fig. S4. Western blot analysis of LPG in *cpct* ⁻ mutants. Log phase and stationary phase (day 1 and day 3) promastigotes were washed once in PBS and resuspended at 5.0 × 10^7 cells/ml in SDS sample buffer. After SDS-PAGE and transfer, blots were probed with antibodies against LPG (**A**) or α-tubulin (**B**), followed by HRP-conjugated secondary antibodies. Six independent experiments were performed and one representative image is shown here.

Table S1. Summary results of randomly selected *cpct*⁻/+GFP-CPCT cells labeled with anti-*T. brucei* BiP antibody (ER marker). Images were analyzed by the JaCOP Image J software and the Pearson correlation coefficient (PCC) between the localizations of BiP and GFP was determined. A complete overlap is 1.00 and no overlap is 0. Average of 8 images (30 cells) = 0.85. Standard deviation = 0.056.

Figure S1A

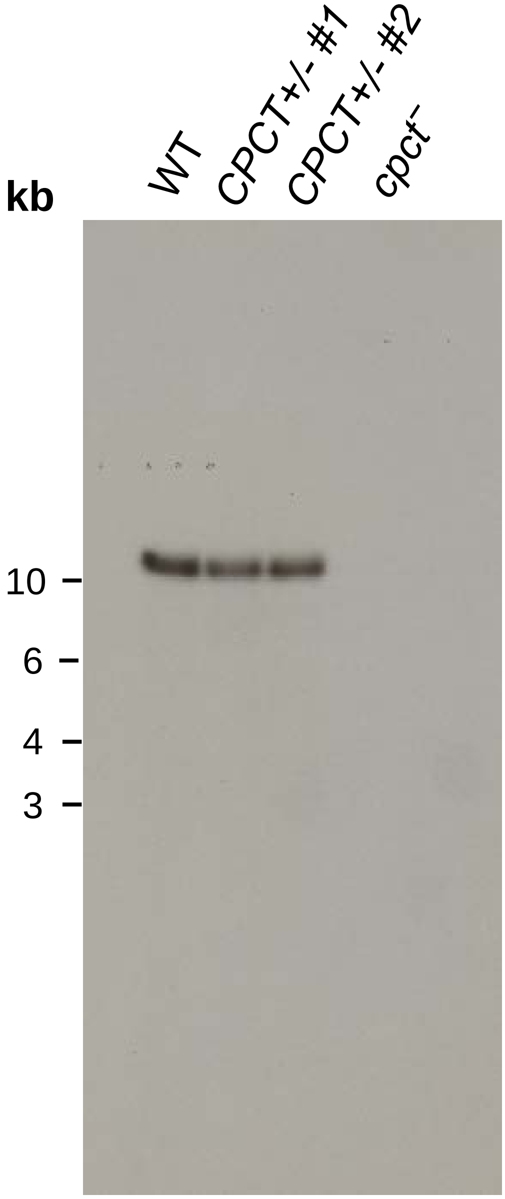

Figure S1B

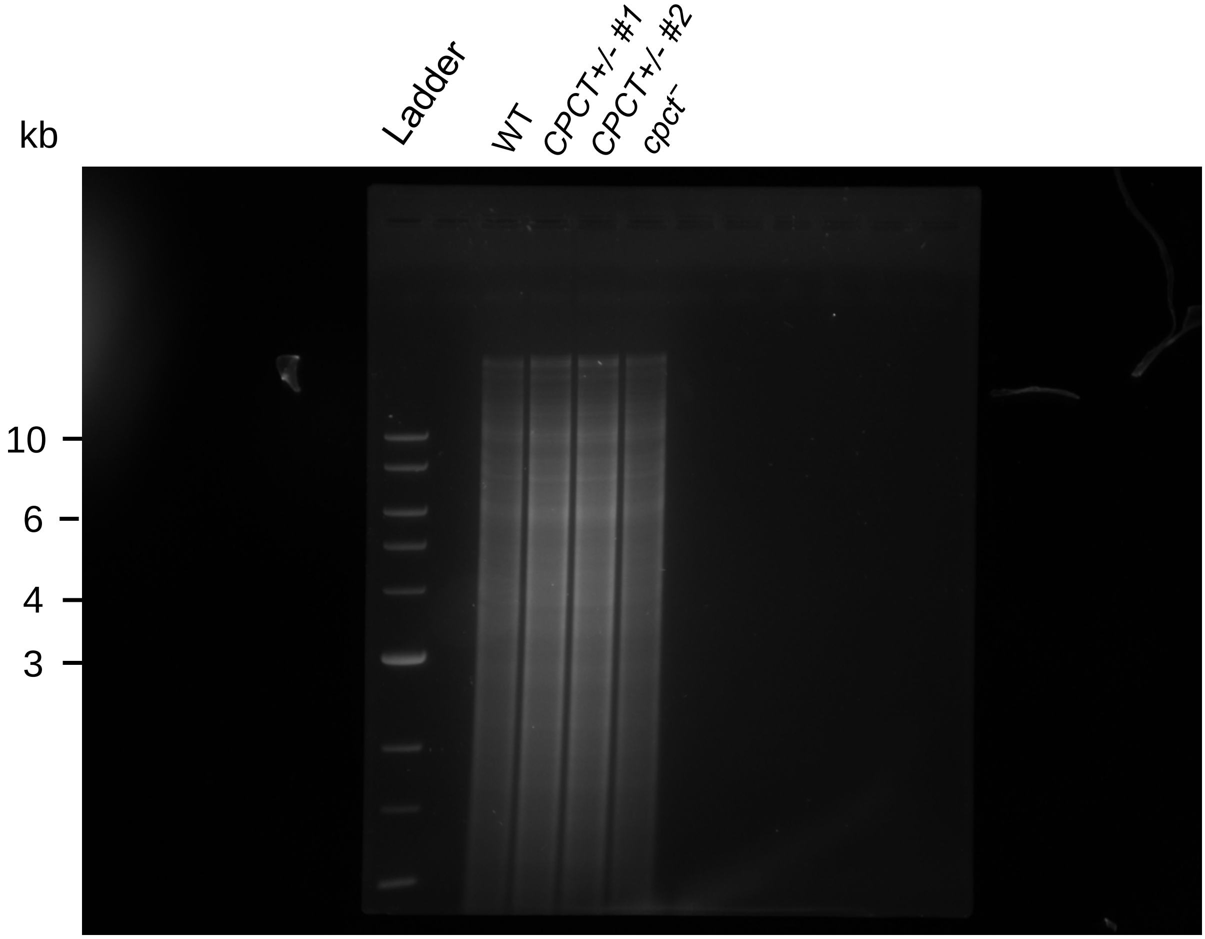

Figure S1C

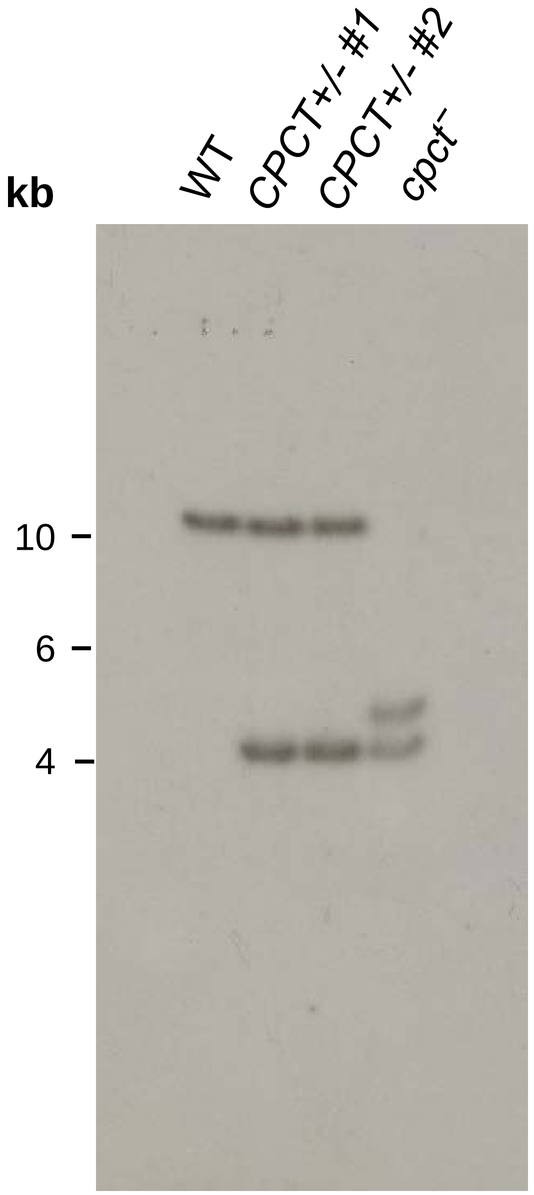

Figure S1D

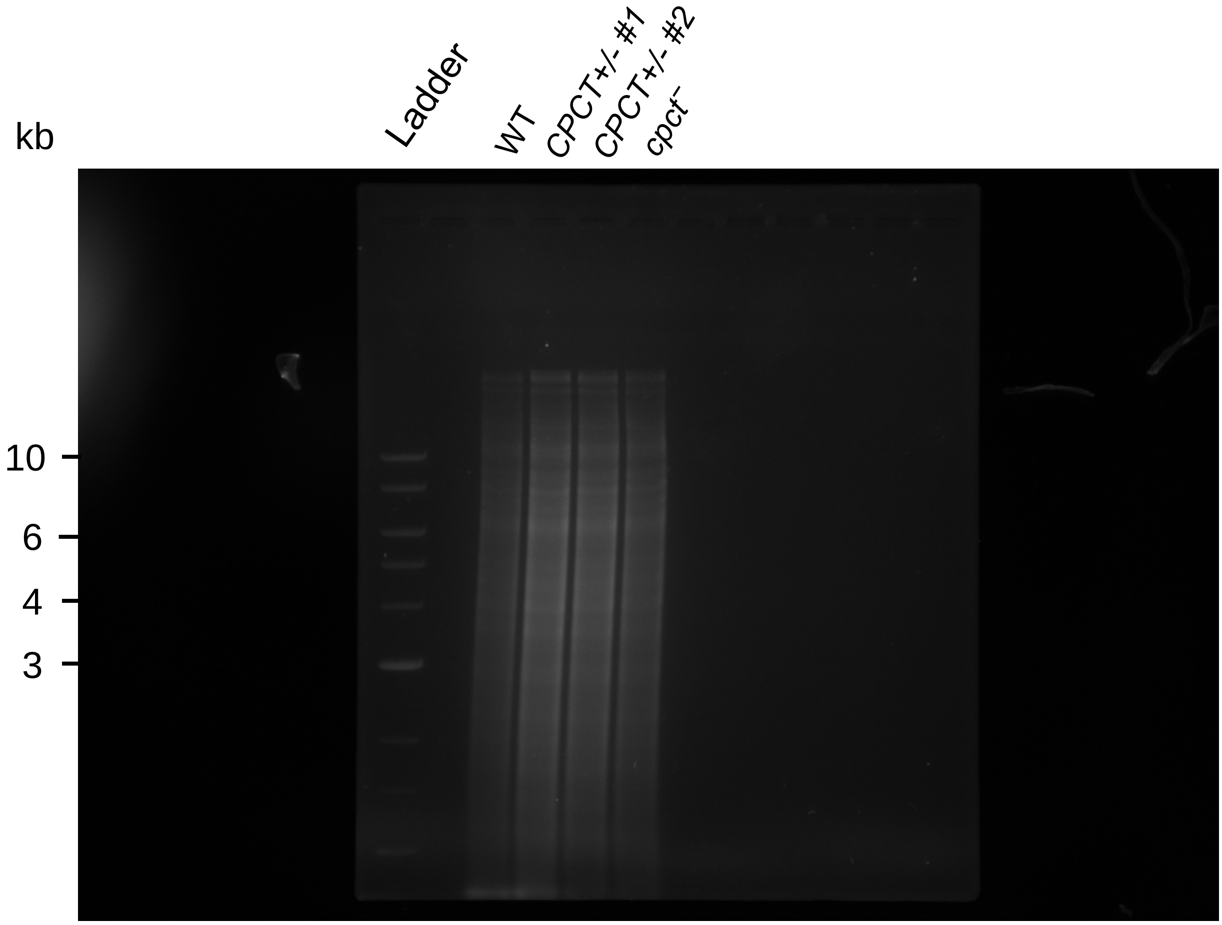

Figure S2A

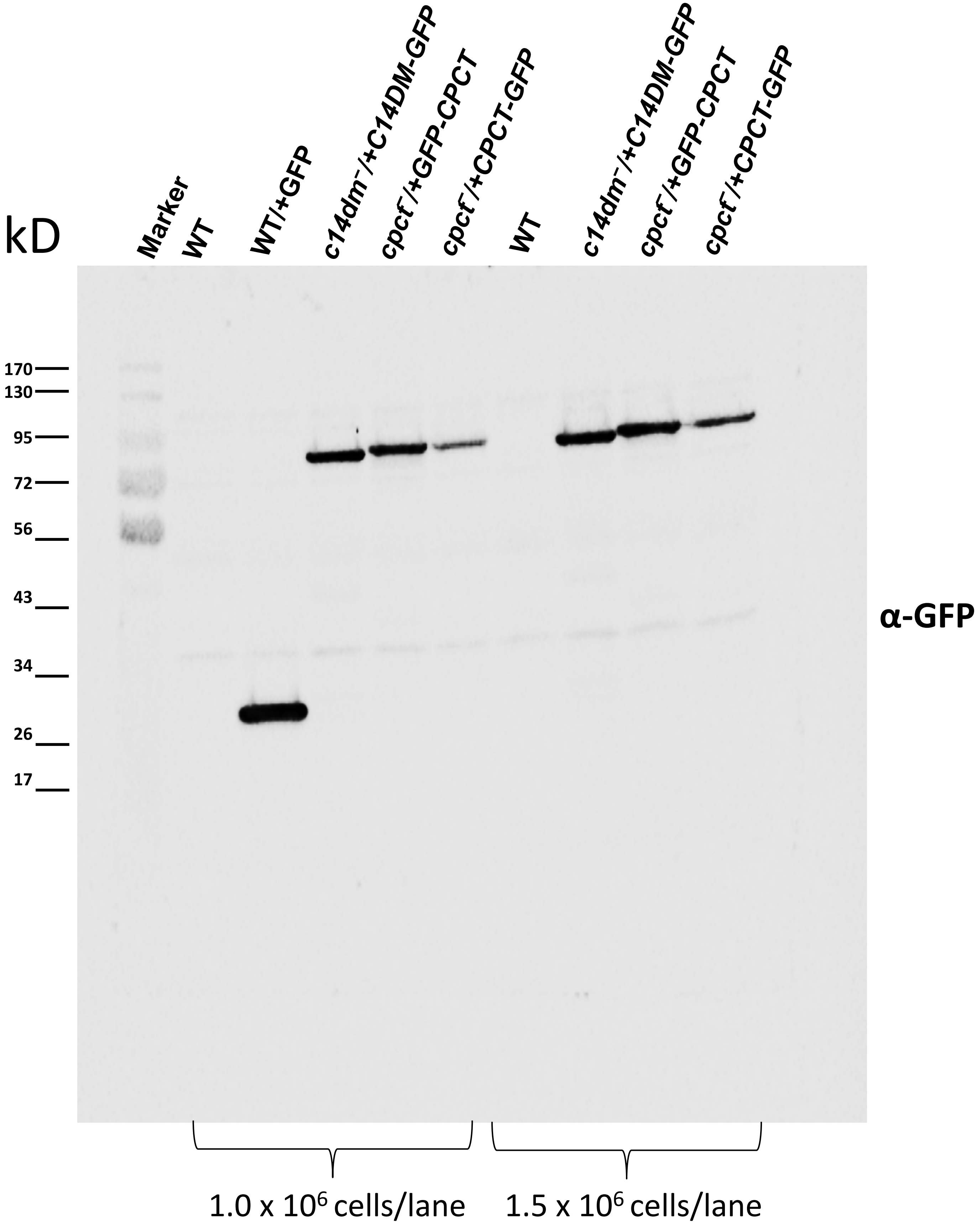

kD
Marker
WT
WT/+GFP
c14dm⁻/+C14DM-GFP
cpcf⁻/+GFP-CPCT
cpcf⁻/+CPCT-GFP
WT
c14dm⁻/+C14DM-GFP
cpcf⁻/+GFP-CPCT
cpcf⁻/+CPCT-GFP
170
130
95
72
56
43
34
26
17
α-GFP
1.0 x 10^6 cells/lane
1.5 x 10^6 cells/lane

Figure S2B

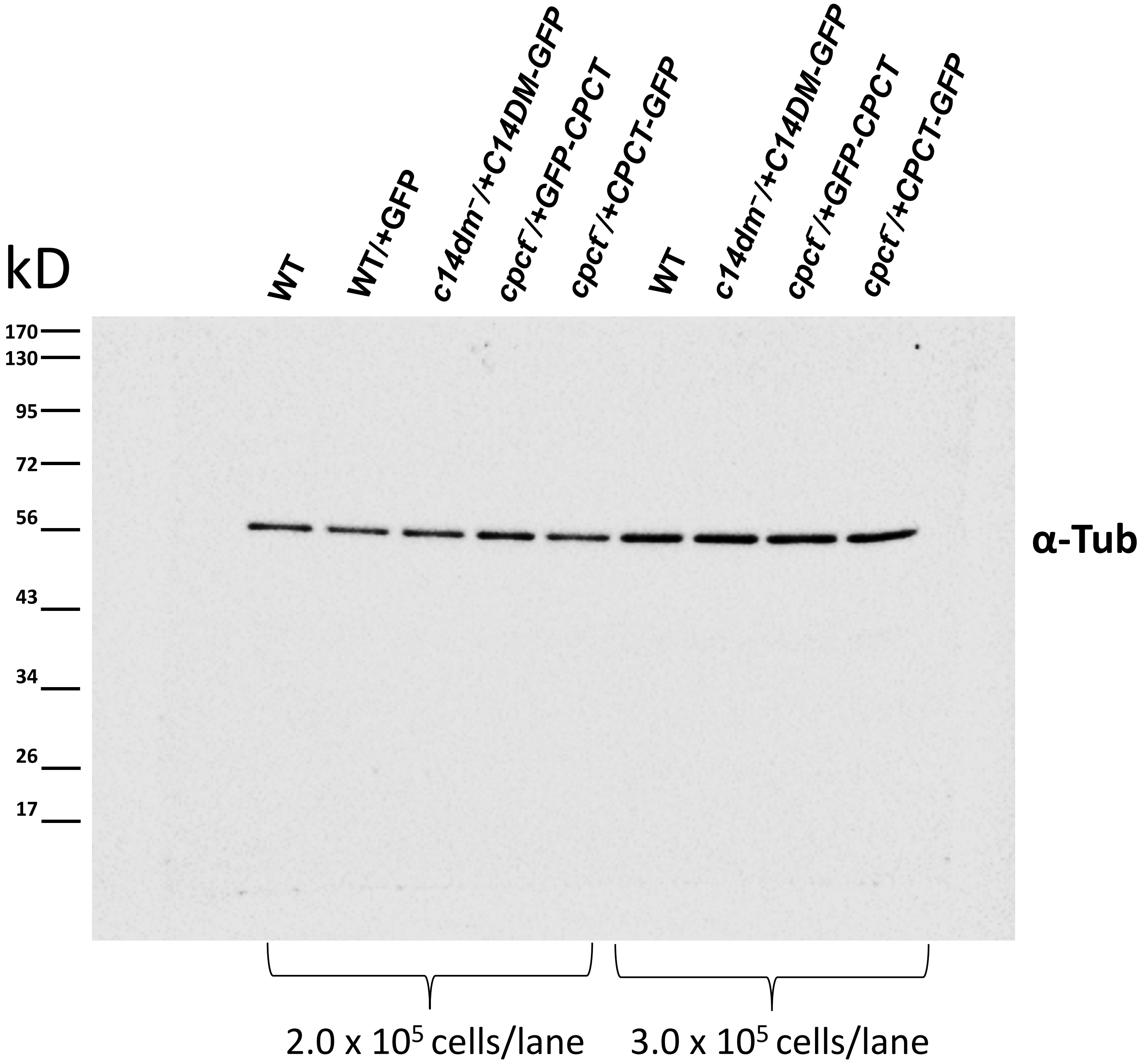
kD
WT
WT/+GFP
c14dm⁻/+C14DM-GFP
cpcf⁻/+GFP-CPCT
cpcf⁻/+CPCT-GFP
WT
c14dm⁻/+C14DM-GFP
cpcf⁻/+GFP-CPCT
cpcf⁻/+CPCT-GFP
170
130
95
72
56
43
34
26
17
α-Tub
2.0 x 10^5 cells/lane
3.0 x 10^5 cells/lane

Figure S3A

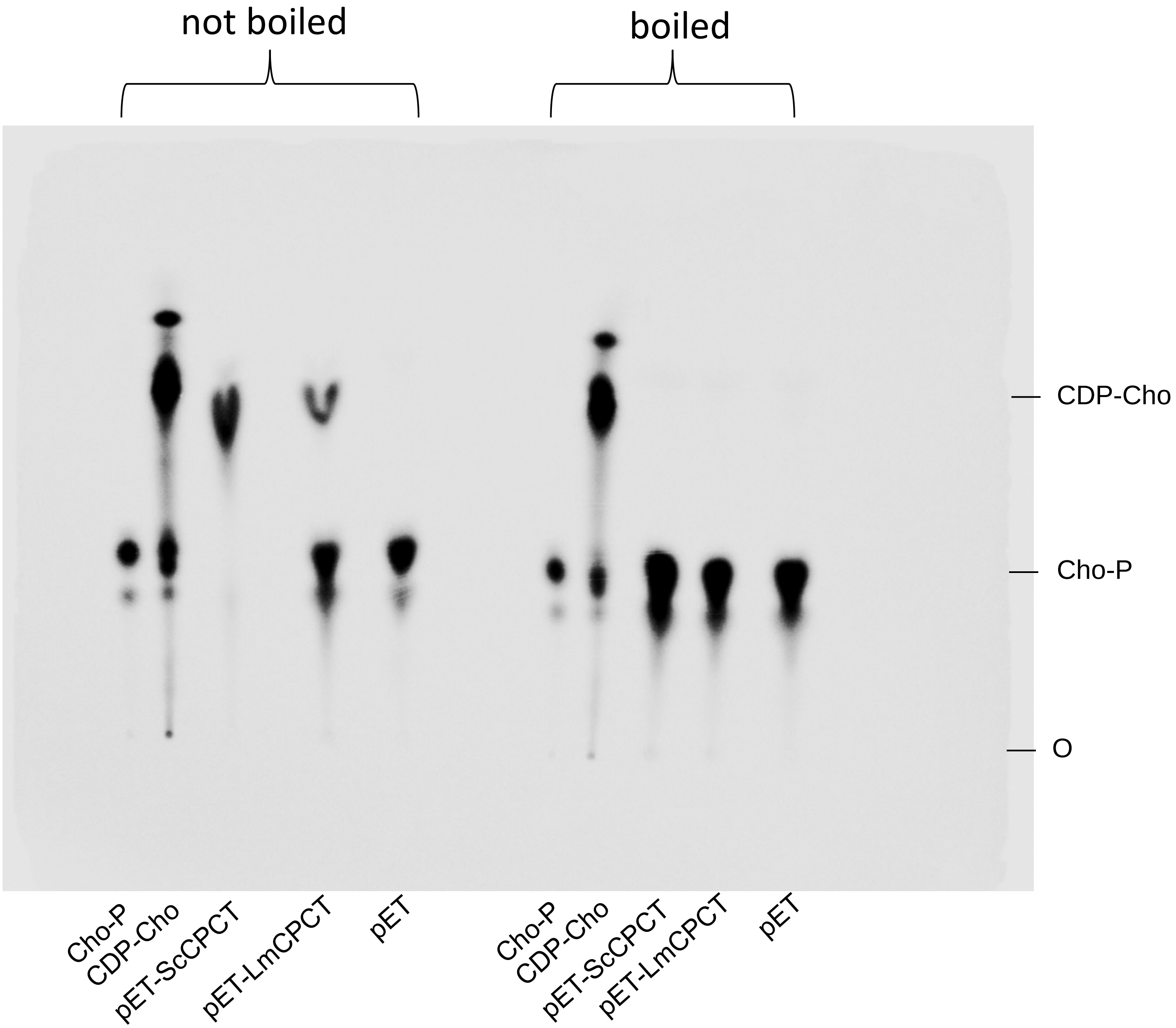
not boiled
boiled
CDP-Cho
Cho-P
O
Cho-P
CDP-Cho
pET-ScCPCT
pET-LmCPCT
pET
Cho-P
CDP-Cho
pET-ScCPCT
pET-LmCPCT
pET

Figure S3B

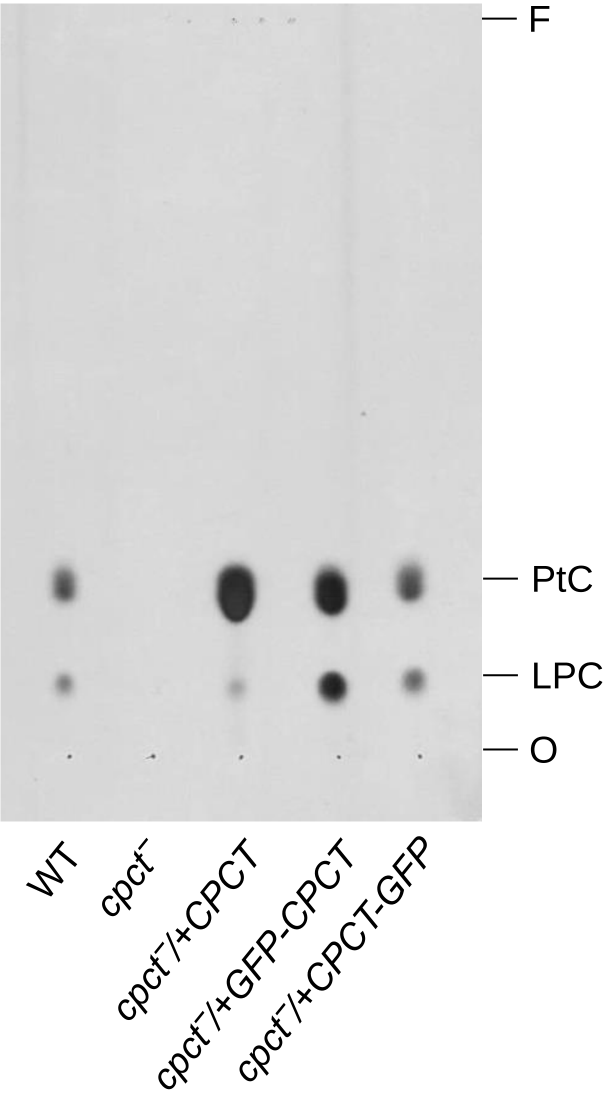

Figure S3C

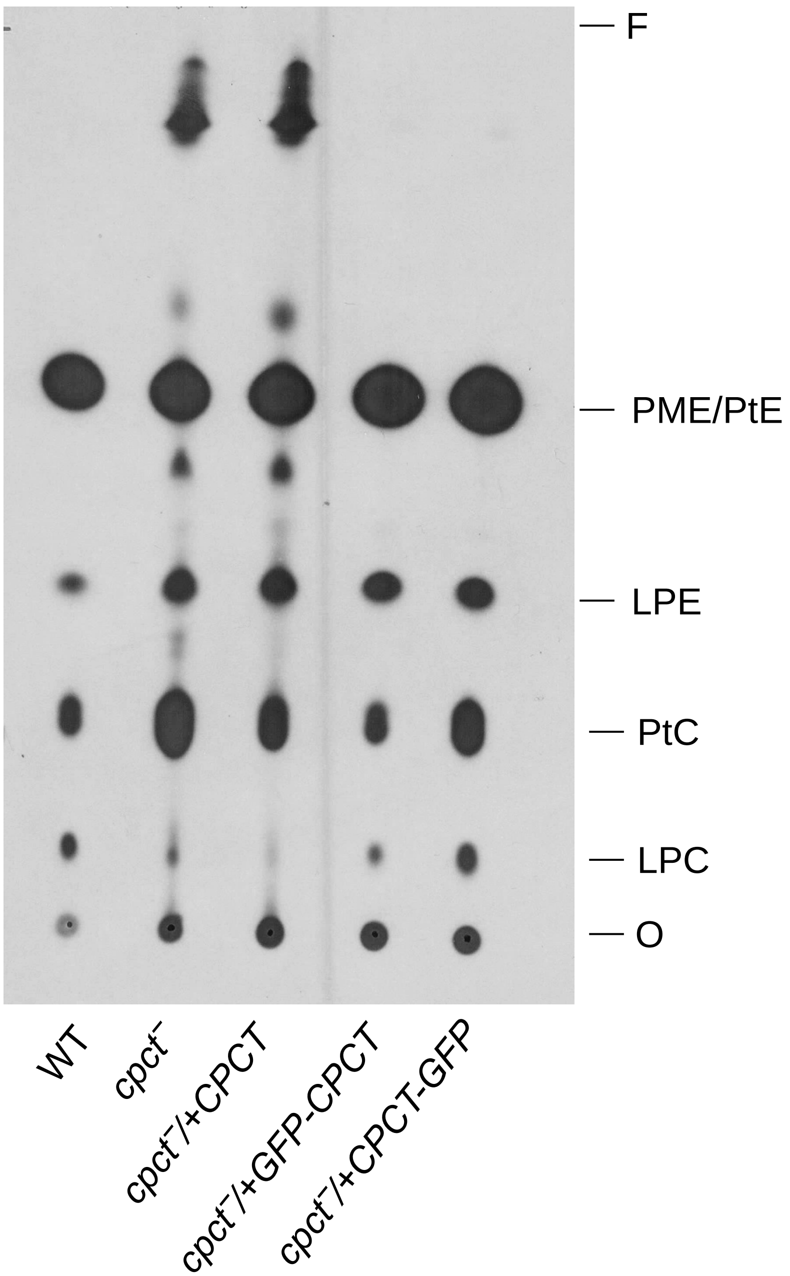
F
PME/PtE
LPE
PtC
LPC
O
WT
cpct⁻
cpct⁻/+CPCT
cpct⁻/+GFP-CPCT
cpct⁻/+CPCT-GFP

Figure S4A

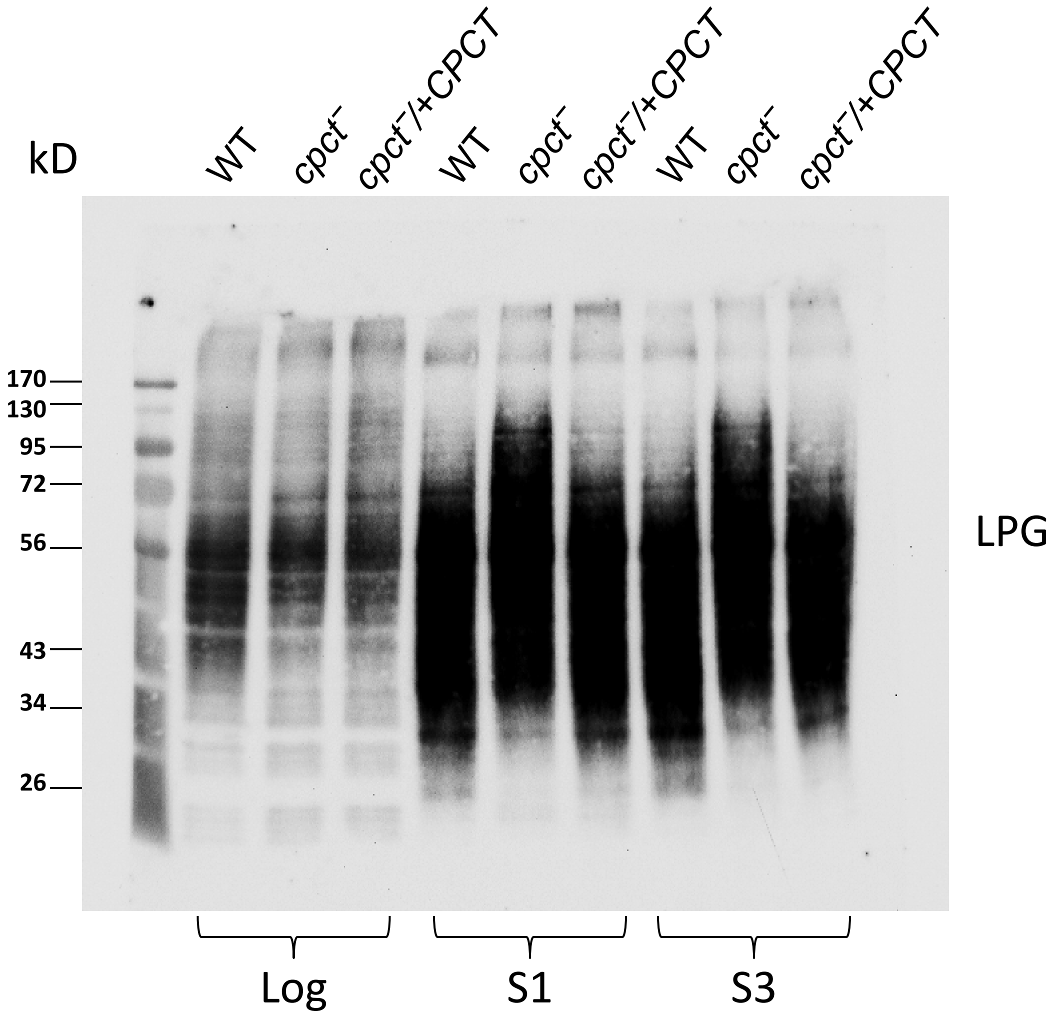

kD
WT
cpct⁻
cpct⁻/+CPCT
WT
cpct⁻
cpct⁻/+CPCT
WT
cpct⁻
cpct⁻/+CPCT
170
130
95
72
56
43
34
26
LPG
Log
S1
S3

Figure S4B

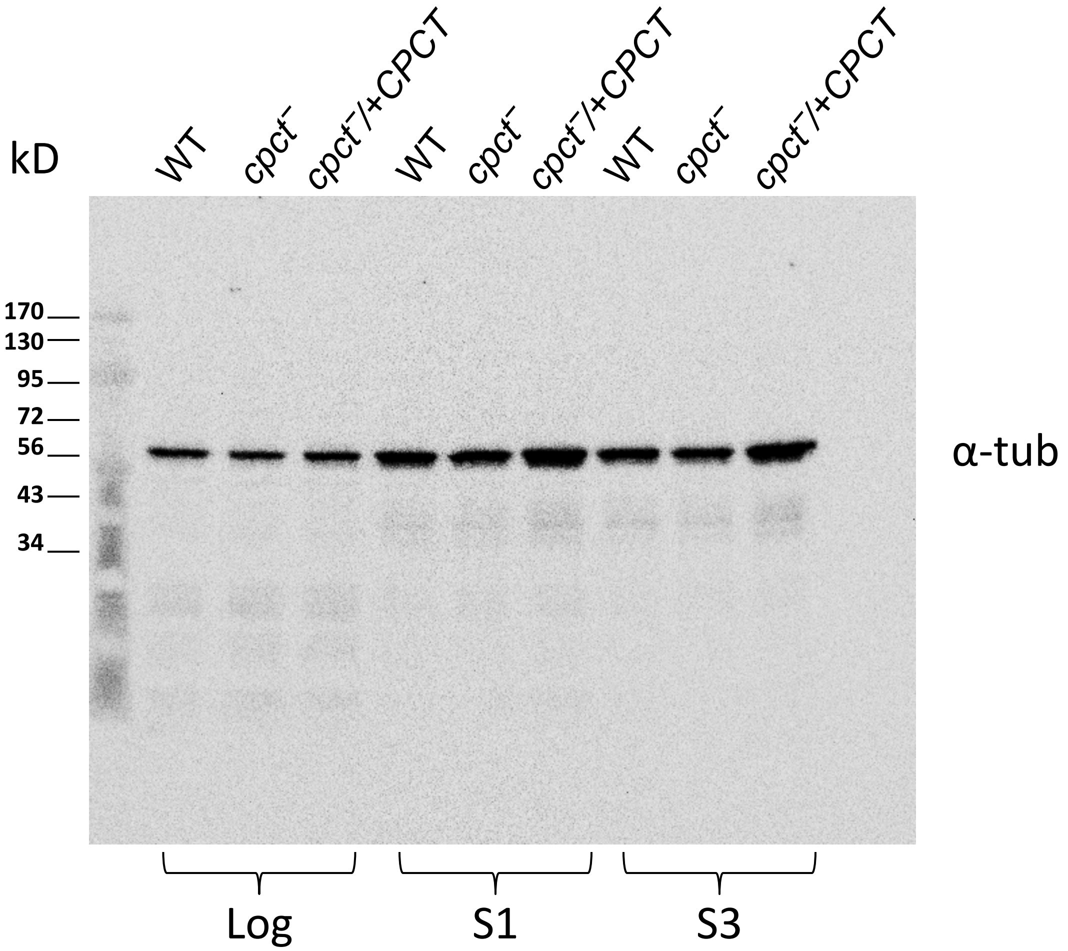

kD
WT
cpct⁻
cpct⁻/+CPCT
WT
cpct⁻
cpct⁻/+CPCT
WT
cpct⁻
cpct⁻/+CPCT
170
130
95
72
56
43
34
α-tub
Log
S1
S3

Table S1. Summary results of randomly selected *cpct*¯/+GFP-CPCT cells labeled with anti-*T. brucei* BiP antibody (ER marker). Images were analyzed by the JaCOP Image J software and the Pearson correlation coefficient (PCC) between the localizations of BiP and GFP was determined. A complete overlap is 1.00 and no overlap is 0. Average of 8 images (30 cells) = 0.85. Standard deviation = 0.056.

Image #	PCC
1 (average of 3 cells)	0.80
2 (average of 5 cells)	0.76
3 (average of 2 cells)	0.89
4 (average of 6 cells)	0.84
5 (average of 2 cells)	0.86
6 (average of 6 cells)	0.87
7 (average of 2 cells)	0.94
8 (average of 4 cells)	0.83
